# Climate change, riverine flood risk and adaptation for the conterminous United States

**DOI:** 10.1088/1748-9326/ac1bd7

**Published:** 2021-08-31

**Authors:** Cameron Wobus, Jeremy Porter, Mark Lorie, Jeremy Martinich, Rachel Bash

**Affiliations:** 1 Lynker Technologies, Boulder CO USA 80303; 2 First Street Foundation, Brooklyn, NY USA 11201; 3 Abt Associates, Boulder CO USA 80301; 4 US Environmental Protection Agency, Washington DC USA 20460

## Abstract

Riverine floods are among the most costly natural disasters in the United States, and floods are generally projected to increase in frequency and magnitude with climate change. Faced with these increasing risks, improved information is needed to direct limited resources toward the most cost-effective adaptation actions available. Here we leverage a newly available flood risk dataset for residential properties in the conterminous United States to calculate expected annual damages to residential structures from inland/riverine flooding at a property-level; the cost of property-level adaptations to protect against future flood risk; and the benefits of those adaptation investments assuming both static and changing climate conditions. Our modeling projects that in the absence of adaptation, nationwide damages from riverine flooding will increase by 20–30% under high levels of warming. Floodproofing, elevation and property acquisition can each be cost-effective adaptations in certain situations, depending on the desired return on investment (i.e., benefit cost ratio), the discount rate, and the assumed rate of climate change. Incorporation of climate change into the benefit-cost calculation increases the number of properties meeting any specified benefit-cost threshold, as today’s investments protect against an increasing frequency of future floods. However, because future expected damages are discounted relative to present-day, the adaptation decisions made based on a static climate assumption are very similar to the decisions made when climate change is considered. If the goal is to optimize adaptation decision making, a focus on quantifying present-day flood risk is therefore at least as important as understanding how those risks might change under a warming climate.

## 1. Introduction

Coastal and inland flooding cause billions of dollars in damage in the United States every year (e.g., Downton and Pielke, 2005; Gall et al., 2011; NOAA, 2021), and there is evidence that flood damages have increased in recent decades (e.g., Grimm, 2020). Furthermore, it is very likely that a warming climate will increase flood damages as storms increase in both frequency and severity (e.g., Wobus et al., 2017; Wing et al., 2018). Adapting our built infrastructure to this changing risk will be one of the most pressing challenges of our generation. To do this, however, we will need robust models to describe current and future climate risks so that limited resources can be prioritized and invested in targeted adaptation actions. Until recently, the data required to prioritize adaptation investments at local scales have not been available at a sufficiently granular scale to inform these decisions. In addition, riverine flood maps from FEMA are typically available for only one or two recurrence intervals (typically the “100-year” and “500-year” flood events), which does not allow calculation of the expected annual damages (EAD) from floods of all magnitudes (Ward et al., 2011; Wobus et al., 2019). Finally, most publicly available flood risk products do not sufficiently account for the impact of climate change on the future magnitude and frequency of floods.

In this paper, we leverage a new flood risk dataset for the conterminous United States (Bates et al., 2021; Armal et al., 2020; First Street 2020a, First Street, 2020b) to conduct a screening-level assessment of current and future flood risk, the costs of property-level flood adaptation, and the benefits of those adaptations under current and future climate regimes. We focus on three specific types of flood adaptation – floodproofing, elevation, and buyout/demolition – because each of these can be implemented at a property level, and because each of them can be subsidized in the United States if they can be shown to be cost-effective (FEMA, 2015). We frame our discussion in purely economic terms, assuming that adaptation decisions are made rationally by weighing today’s adaptation costs against a future stream of benefits in the form of avoided flood damages. These future benefits are discounted to present value to account for the time value of money. We then evaluate the sensitivity of these decisions to factors including the assumed discount rate, the desired benefit-cost ratio (i.e., return on investment), and whether or not changing flood risk due to climate change is incorporated into the calculation of future avoided damages. Although we do not explicitly address the components of human behavior that complicate these decisions (e.g., Harries, 2012; Grothmann and Patt, 2005), our framework provides an important first step toward quantifying the national-scale costs and benefits of riverine flood adaptation at a property level.

Our methodology is as follows: First, we use the expected annual damage (EAD) from flooding at each property in the United States as a metric of riverine flood risk under today’s climate. Using flood exposure information along with actual and imputed building size and construction type data (e.g., Armal et al., 2020), we then estimate the cost of adaptation for each property using simple decision rules to determine what type of adaptation is most appropriate for each property. This allows us to perform a simple benefit cost test for each property to determine which simulated adaptation investments may be cost-effective, under today’s climate conditions.

We then evaluate how climate change might alter adaptation decision-making at a property level. Using publicly available hydrologic projections (e.g., Reclamation, 2014; Wobus et al., 2017), we calculate the future frequency of floods with present-day return intervals ranging from 2 years through 500 years, under scenarios of CONUS-averaged temperature increases ranging from 1°C through 5°C warmer than baseline. These flood frequency adjustments create changes in EAD for each property under each of those temperature scenarios or thresholds. We propagate those changes in EAD through the benefit-cost framework to evaluate how today’s adaptation decisions might be adjusted to account for a warming climate.

Our analysis builds on existing data (e.g., Davenport et al., 2021; First Street, 2020a) that illustrate which parts of the United States have the highest levels of economic exposure to inland flooding under current and multiple future climate scenarios. In addition, our property-level benefit-cost framework provides a mechanism to assess where adaptation investments could be prioritized, and how climate change informs adaptation decisions to mitigate riverine flood risk.

## 2. Data and Methods

We based our analysis on a dataset that was developed as part of an open-source, parcel-level flood risk model for the United States (First Street Foundation, 2020a; Bates et al., 2021). For each parcel in the conterminous United States, this dataset includes the primary driver of flood risk (i.e., coastal, fluvial or pluvial), as well as the projected depth of flooding for return intervals of 2 years (50% annual exceedance probability, or AEP) through 500 years (0.2% AEP). The dataset also includes an estimate of the first floor (ground-level) elevation for each building, the building type, the square footage, the replacement cost, and the market value for each property. Details regarding the development of this dataset are available in First Street Foundation (2020a) and Armal (2020). Using this dataset, we calculated the EAD for each property under baseline climate conditions, as follows.

**Figure 1.**
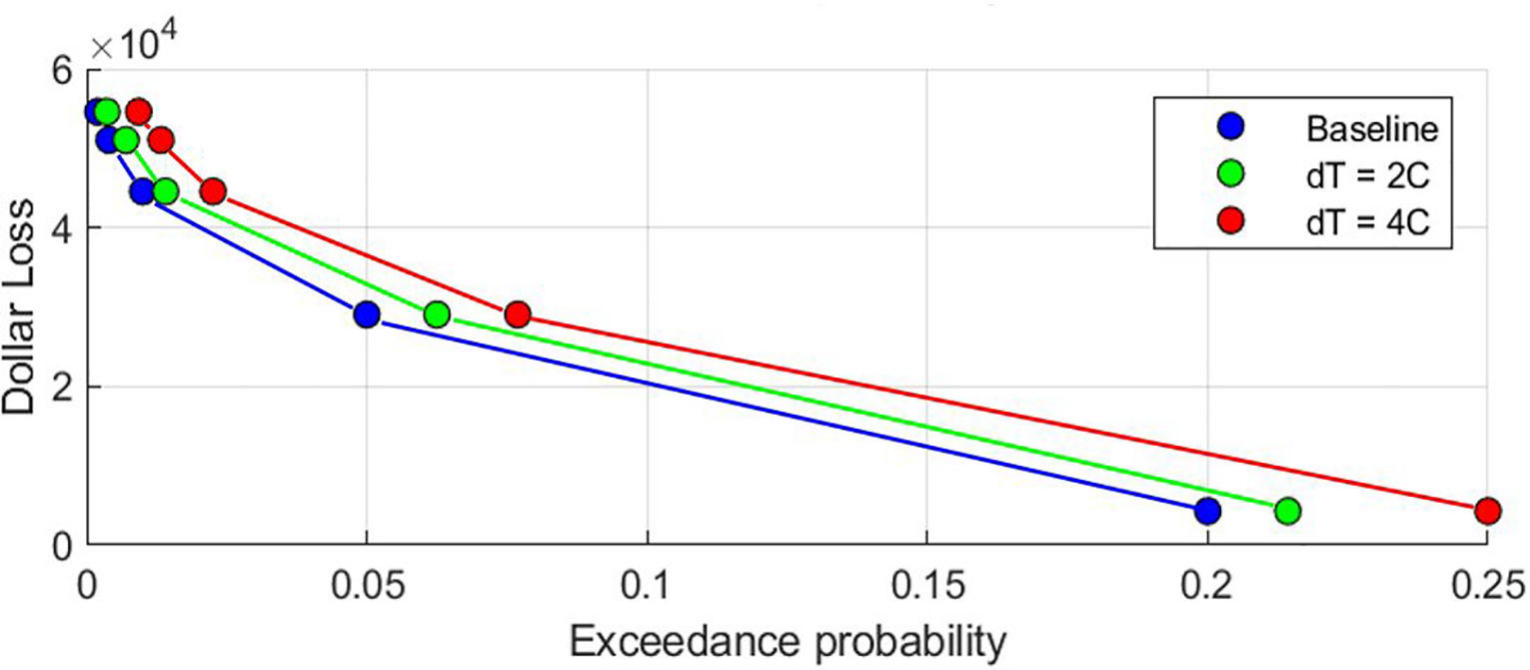
Schematic illustrating the calculation of EAD for baseline (blue) and future climate conditions (green and red). Discrete dollar losses are calculated from flood depths with probabilities of 0.2, 0.05, 0.01, 0.004 and 0.002. Losses were interpolated between those intervals and total area under the curve was calculated between 0.1 and 0.001. Changing probabilities for future events increase or decrease the area under the curve and therefore the EAD.

The first step was to subtract the first-floor elevation from each flood depth value, to obtain the depth of flooding relative to the first floor at each recurrence interval. Using each of these flood depths, we then calculated the fractional loss that would be incurred to the property due to a flood with that recurrence interval. These fractional losses were determined by matching the occupancy class for each property with the appropriate depth-damage curve, following the guidelines in the HAZUS manual to select a unique depth-damage curve for each occupancy class (FEMA, n.d.). Using the combination of fractional loss, building replacement value, and recurrence interval, we then constructed a frequency-loss curve for each property and numerically integrated under this curve between flood frequencies of 0.001 and 0.10 to calculate the EAD (Figure 1).

### 2.1 Adaptation

For each parcel, we evaluated three adaptation actions to mitigate riverine flood risk: dry floodproofing, elevation, and property acquisition/buyout. Using guidance from FEMA (2009), we established simple rules based on the current 1% AEP flood depth to evaluate which adaptation action would be most appropriate for each parcel based on the practical limits and costs of each adaptation. We assumed that dry floodproofing would be favored if the depth of flooding for a 1% AEP event is between 0–3 ft; that elevation would be favored if the depth of flooding is between 3–8 ft; and that property acquisition would be favored if the flood depth is greater than 8 ft. For dry floodproofing and elevation, we assumed that protection would be built to the base flood elevation plus 1 ft of freeboard.

We acknowledge that these simple rules do not capture all of the property-specific complexities that would drive response decisions on the ground. For example, we did not optimize adaptation decisions based on BCR, nor did we consider combinations of adaptation measures at a single property. We also do not have the detailed property-level information required to evaluate what adaptations may already be present at an individual parcel. All of these site-specific considerations are beyond the scope of our national-scale study; however, communities or individual property owners could adapt our framework to incorporate these site-specific elements into their individual decision-making.

For each adaptation action, we combined unit costs of adaptation with available data on total building square footage (First Street Foundation, 2020a; Armal, 2020). We combined this information with the number of floors for each building to calculate the size of each building footprint, on which unit costs for property elevation are based. For floodproofing, we used the square footage of the building footprint and an assumed building length:width ratio of 2:1 to estimate the building perimeter, on which unit costs for floodproofing are based.

#### Dry Floodproofing

In a dry floodproofing approach, the livable area that would be exposed to flood water is made watertight to eliminate the potential of the space being inundated in a flood situation. We assume dry floodproofing is achieved with permanent watertight coating on exterior walls of the structure. The watertight coating can be applied to above-ground exterior walls up to three feet above ground level. Flooding above three feet of inundation depth creates hydrostatic pressure that many structures may not be able to withstand (FEMA, 2009) and it may start to reach the height of window sills, which are difficult to seal. Furthermore, FEMA (2009) indicates that dry floodproofing is generally not appropriate for homes with basements, due to the high potential for foundation walls to fail under increased hydrostatic pressure when flooded. We assume dry floodproofing is feasible for any structure that does not have a basement, and that this adaptation will eliminate flood damage for depths up to three feet above ground.

Unit costs for dry floodproofing depend on the specific combination of techniques chosen. Table S1 shows unit costs for several alternatives (FEMA, 2009, adjusted to 2020 dollars). We include the following measures from FEMA (2009): 1) waterproof membrane, above grade only, 2) drainage lines around the perimeter of the structure, 3) one plumbing check valve per structure, 4) one sump pump per structure, and 5) six linear feet of metal flood shield per 1000 square feet of footprint. As an example, dry floodproofing for a single story house with a 130 foot perimeter would total approximately $13,500.

#### Elevation

Elevating a home or structure involves lifting the structure above a design flood elevation. This is accomplished either by lifting the entire foundation and structure (for slab-on-grade foundations) or by extending foundation walls vertically (typically for structures with a basement or crawl space). We assume that elevation is feasible for homes with or without basements. For homes with slab-on-grade construction, we assume that elevation will be implemented to protect against floods with more than 3 ft of inundation (i.e., beyond the limits of dry floodproofing). For homes with basements, we evaluate elevation as an option for any floods above the first floor elevation.

Unit costs for elevation were taken from FEMA (2009), a source widely used to prepare large regional adaptation cost estimates (e.g., see Botzen, 2013, Aerts, 2018). FEMA (2009) provides unit cost values for three increments of elevation. From these data, we calculated a fixed cost for the initial 2 ft of elevation (covering design, regulatory requirements, and mobilization of construction equipment) and an incremental cost for each successive foot of elevation beyond the first two feet. Per FEMA (2009), elevation requires less structural work for buildings with basements than for those on a slab foundation. We incorporated these differences into our analysis, as shown in Figure S1.

#### Property Acquisition (Buyout)

For properties with more than 8 feet of flooding in the 1% AEP event, we assume that structural solutions like floodproofing and elevation become impractical. For those properties, we assume that property acquisition is the most appropriate adaptation decision. We further assume that the acquisition cost would be the market value of the property, which is included in the property-level database (e.g., First Street Foundation, 2020a; Armal et al., 2020).

### 2.2 Benefit-Cost Framework

Once simulated adaptations are selected based on projected flood depth, we calculate the benefit-cost ratio for each adaptation action. The annual projected benefit from each adaptation is the reduction in EAD due to adaptation. For dry floodproofing, the reduction in EAD is due to the elimination of damage from any flood events smaller than the current 1% AEP flood, up to three feet above the first floor elevation. For floods larger than the flood protection depth, we assume that damages would be the same as before adaptation, as floodwaters are assumed to overtop the first floor windows. For elevation, the residual damage from events larger than the flood protection depth is based on the flood depth after elevation. Thus for a property elevated 4.5 feet, the modified flood depth from a flood 6 ft above the previous first floor elevation becomes 1.5 ft, shifting the fractional loss along the depth-damage curve accordingly.

To compare the benefits and costs of each action, the cost of adaptation today is compared to the change in net present value of EAD discounted over 30 years at 3%. For simplicity and transparency, we choose a 30-year timeframe for estimating benefits for all adaptation types. This corresponds to the timeframe of a typical home mortgage, and is therefore a timescale relevant to individual homeowner decision-making. For climate-related analyses, the 3% discount rate is a conservative choice given considerations of uncertainty in the discount rate over long time horizons (Carleton and Greenstone, 2021), with recent federal guidance advocating for the usage of rates below 3% based on the best available science on intergenerational discounting (IWGSCGG, 2021). We test the sensitivity of our results to higher (6%) and lower (1%) discount rates in the Results.

### 2.3 Climate Change

For this analysis, each future climate scenario or threshold is represented by successively higher CONUS-averaged temperatures relative to a baseline period of 2001–2020. We used the downscaled hydrology dataset developed by Reclamation (2014), which includes daily routed flows at approximately 57,000 stream reaches across the CONUS for an ensemble of 29 global climate models (GCMs). We selected the 14 climate models from this ensemble whose temperature trajectories under RCP 8.5 reach a CONUS-averaged temperature of 5°C above baseline by 2100. RCP 8.5 is a pathway with relatively high greenhouse gas concentrations, leading to substantial warming by 2100, and this selection ensures the evaluation of a broad range of future changes in flood risk.

From each model in the ensemble, we extracted an annual maximum flow time series at each stream reach for a 20-year window centered on the year that the model reaches temperature thresholds of 1°C through 5°C above baseline. Each temperature threshold thus has a set of 280 annual maxima (20 years x 14 GCMs), to which we fit a generalized extreme value (GEV) distribution. For the baseline period of 2001–2020, we extracted from this GEV fit the magnitude of flows corresponding to the 2-year (50% AEP) through 500-year (0.2% AEP) recurrence interval event. For each temperature threshold, we then calculated the future recurrence interval of the flow corresponding to each of these baseline events. We then averaged these future recurrence intervals for all of the stream reaches in each HUC10, allowing us to shift the EAD curve for each property as shown schematically in Figure 1.

Additional details on the methods used for extracting future return intervals can be found in Wobus et al. (2017) and Wobus et al. (2019).

## 3. Results

### 3.1 Riverine Flood Risk and Climate

**Figure 2.**
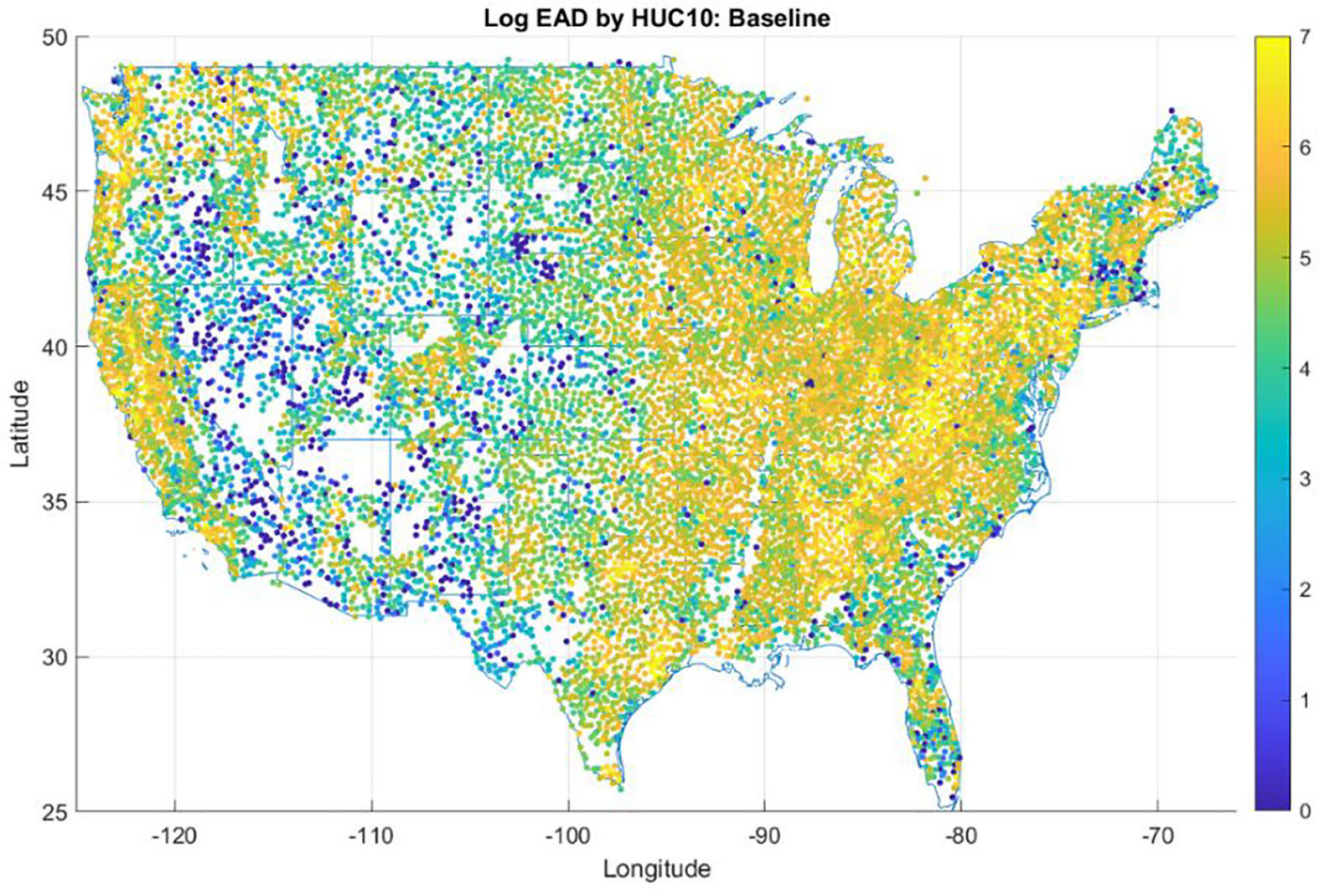
Nationwide distribution of expected annual damages from inland flooding, aggregated by HUC10 watershed. Total EAD for all properties in each HUC10 is shown at the centroid of each watershed. Colorbar is a log scale (e.g., 6 = $1M, 7 = $10M)

Figure 2 shows the spatial distribution of inland flood risk in the United States under baseline climate conditions, expressed as property-level EAD aggregated up to HUC10 watersheds. These spatial patterns of flood risk reflect a combination of property values, population density, and regional differences in the types of storms that produce riverine flooding, such as hurricanes and frontal storms. Based on this analysis, the total EAD from riverine flooding nationwide is approximately $7 billion under current climate, which represents approximately 1/3 of the average annualized loss reported by First Street Foundation (2021) for all flooding types (including coastal and pluvial).

**Figure 3.**
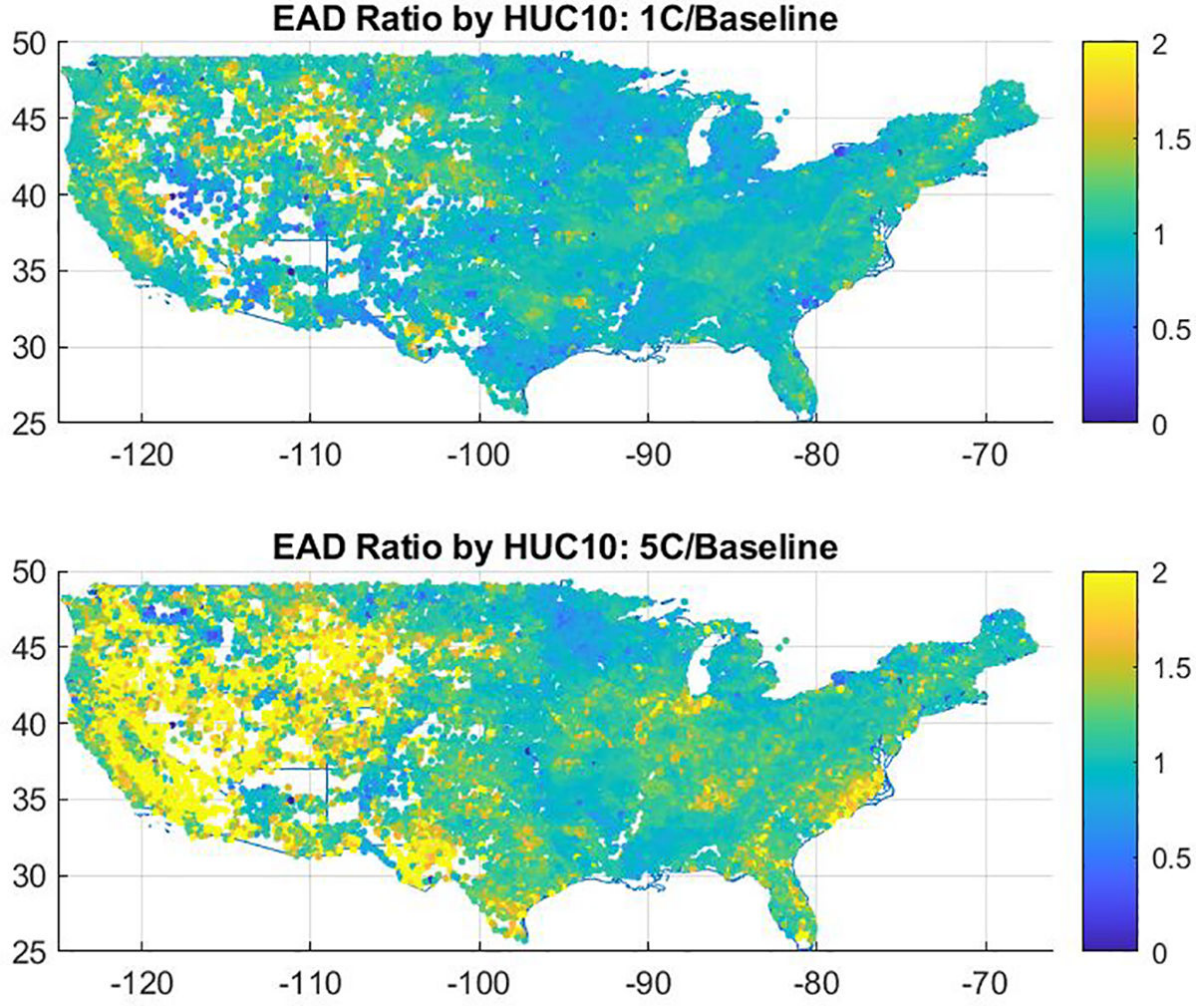
Spatial distribution of proportional changes in EAD from riverine flooding due to temperatures of A) 1°C and B) 5°C above baseline. EAD changes are aggregated by HUC10 watershed, and shown at the centroid of each. Values of 2 represent a doubling of damages by HUC10, and values of ½ represent a halving of damages.

Figure 3 shows the spatial distribution of changes in EAD for CONUS averaged temperature changes of 1°C and 5°C warmer than the baseline period. As shown, the largest proportional changes in EAD occur in the western United States, the Southeast, and the Midwest, and these changes are especially pronounced at higher levels of warming. Nationwide, the total EAD increases by approximately 30% - from approximately $7B in the baseline to approximately $9B in a 5°C warmer CONUS.

### 3.2 Adaptation Decisions for Inland Flooding

**Figure 4.**
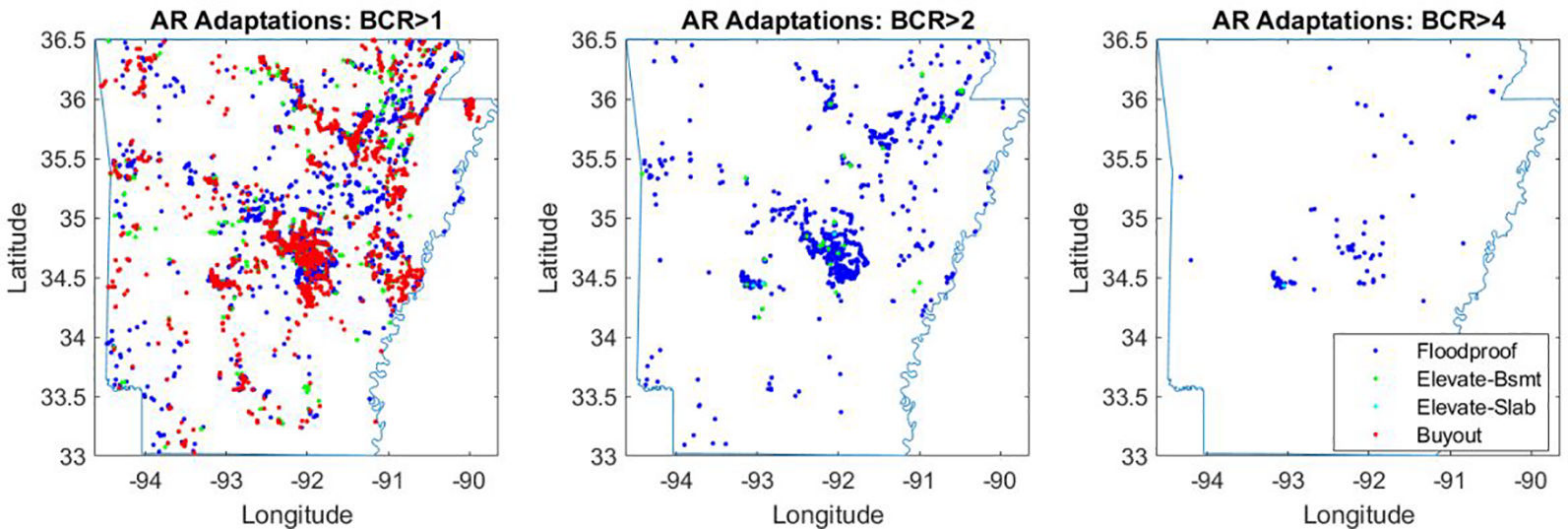
Example of how property-level adaptation decision-making changes with BCR, for the state of Arkansas. As the BCR threshold increases, the number of properties meeting that threshold decreases, and buyout and elevation become less likely to pass the BCR test.

For each property, we compare the cost of adaptation in today’s dollars against the net present value of the reduction in EAD due to adaptation. In other words, the reduction in EAD represents a stream of future benefits that is discounted to today’s dollars. This comparison yields a benefit-cost ratio (BCR), which is then used to guide simulated decisions at an aggregate level. As an example, Figure 4 shows the number of properties that meet benefit-cost ratios of 1, 2, and 4, for the state of Arkansas. Note that while the property-level flood depths in the baseline dataset include existing flood protection infrastructure like levees and dikes (e.g., Bates et al., 2021), we do not consider changes to this existing infrastructure in our analysis.

Available evidence suggests that property owners invest in flood risk reduction less than we would expect from a strict cost-benefit standpoint (Bakkensen and Mendelsohn, 2016; Javeline and Kijewski-Correa, 2019). Our use of multiple BCRs is meant to approximate the impact of sub-optimal decisions among property-owners (e.g., Lorie et al, 2020), and to provide an estimate of the return on investment for those decisions. In the Arkansas example shown in Figure 4, for a BCR of 1, the results show a blend of floodproofing, elevation, and buyouts across the state. As the benefit cost threshold increases, both the number and the types of adaptation change. For example, while approximately 13,000 properties in Arkansas meet a BCR of 1, only ~2,000 properties meet a BCR of 2, and none of these properties exceed this threshold for buyout. When the BCR threshold is increased to 4, only approximately 100 properties meet this threshold and virtually all of the cost-effective property adaptations that remain are floodproofing.

As described above, climate change is projected to generally increase the expected annual damages at a property-level throughout the CONUS. This also means that well-designed adaptation investments made today will have an increasing benefit in the future, as those adaptations will protect against increasingly frequent damaging flood events. Because adaptation decisions made today are based on a discounted stream of benefits in the future, however, future climate change is not shown here to exert as much leverage on adaptation decision-making as one might expect.

**Figure 5.**
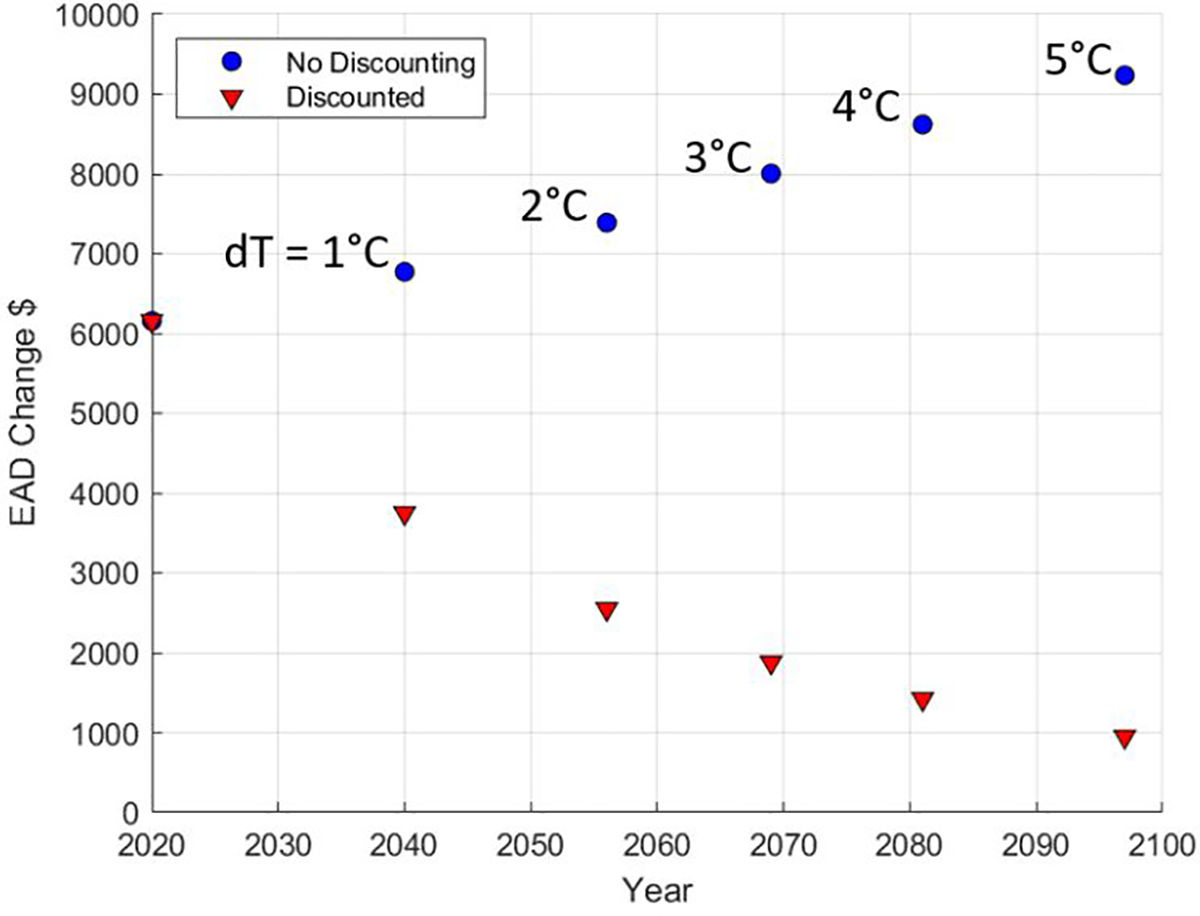
Example of how adaptation changes expected annual damages for a single property. In this example, adaptation protects against successively higher-frequency events with warming (blue circles). However, because those benefits are discounted relative to current benefits, future EAD reductions become less important in the overall benefit cost calculation (red triangles). Arrival times of temperature thresholds are from RCP 8.5, representing a higher emissions scenario.

Figure 5 illustrates this effect for a single property, based on a rapid warming trajectory (RCP 8.5) for CONUS-averaged temperatures. For this property, the benefit of adaptation under baseline climate conditions is a reduction in EAD of approximately $6000 (y-intercept of both curves). This reduction in EAD reflects the elimination of damages from events smaller than the flood protection depth, which decreases the area under the frequency-loss curve. As shown in Figure 5, if the CONUS-averaged temperature were 1°C warmer, the reduction in EAD from that same adaptation investment for this example property would be closer to $7000, and if it were 5°C warmer, the reduction in EAD would be more than $9000 (y values corresponding to blue circles). These larger projected benefits (higher values of EAD change) are accrued because the shift in the frequency-magnitude curve for future floods means that today’s adaptation is protecting this property from a larger number of future floods.

In any adaptation benefit-cost framework, however, today’s adaptation costs must be compared to the *net present value* of future benefits. Even under a high greenhouse gas emissions scenario (RCP 8.5), a CONUS-averaged temperature change of 1°C relative to 2001–2020 is not reached until approximately 2040, and a temperature of 2°C is not reached until approximately 2055. The red triangles in Figure 5 reflect the discounted EAD changes corresponding to each of these temperature thresholds, assuming a 3% discount rate. As shown, the contribution to benefits corresponding to a 1°C warmer climate is discounted to ~$4000, and the contribution to benefits from a 2°C warmer climate is discounted to ~$2500. Thus, the reduction of present-day benefits due to discounting (red triangles) rapidly out-paces the increased EAD change due to climate change (blue circles). Furthermore, because the future stream of benefits is discounted over just 30 years in the benefit cost calculation, the most severe impacts from climate change – which in this example occur at the highest temperature changes – are not even included in the benefit-cost calculation. This suggests that for inland flood risk, the flood adaptation decisions we make based on a benefit cost analysis using historical climate conditions will also be cost-effective in a changing climate.

**Figure 6.**
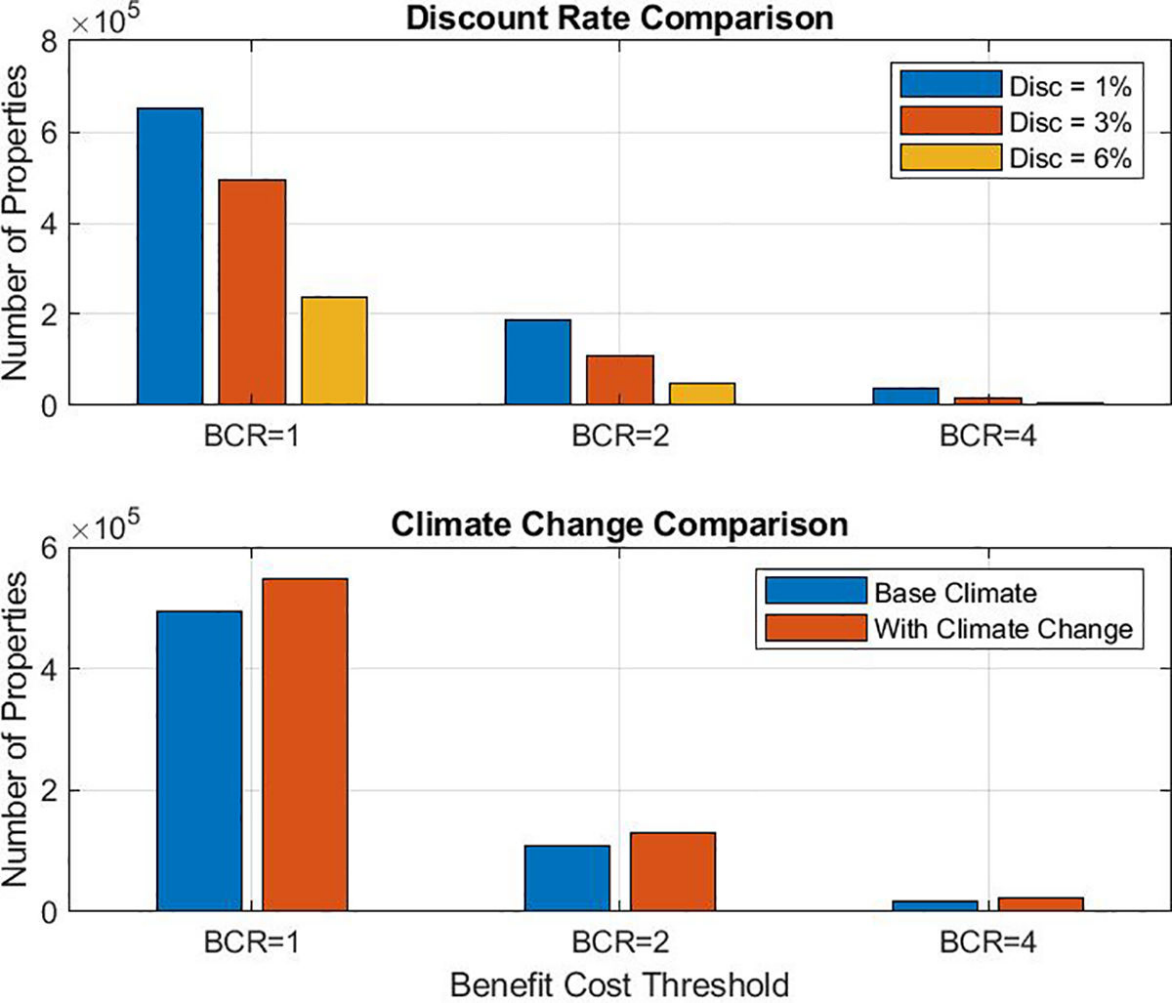
Number of properties exceeding specified benefit-cost ratios (BCR) for adaptation nationwide based on assumptions about discount rate and climate change. A) Number of properties adapted for a given BCR, assuming baseline climate and discount rates of 1%, 3% or 6%. B) Number of properties adapted for a given BCR and a discount rate of 3%, assuming climate change is included (red bars) or is not incorporated (blue bars) in the calculation.

Nationwide, the projected total number of properties to be adapted depends strongly on the choice of benefit cost threshold and discount rate, but is less affected by whether climate change is considered in the response process. As shown in Figure 6, the choice of a benefit cost threshold of 2 vs 1 decreases the number of properties adapted nationwide by more than a factor of three, for a given discount rate. Similarly, choosing a discount rate of 3% vs 1% changes the number of properties adapted by more than 25%, for a given BCR. For a given discount rate and BCR, incorporating climate change into the analysis increases the number of properties adapted nationwide. However, when compared to the influence of BCR or discount rate on the total number of properties adapted, the effect of climate change is relatively small.

The choice of a benefit-cost threshold also has significant implications for how much adaptation investment can reasonably be made to protect against inland flood damages. Although by definition all of the adaptation investments presented in Figure 6 would more than pay for themselves in terms of reduced damages in the future, individuals and governments must also consider the total availability of funding available to commit to these investments. Assuming a 3% discount rate, for example, adapting all of the properties nationwide with a BCR>1 would require a total investment of approximately $46 billion, which would result in a total present value benefit of approximately $69 billion over 30 years if there were no climate change, or approximately $81 billion under a higher emissions scenario (Table 1). On the other end of the spectrum, adapting only the properties nationwide that meet a BCR>4 would require a total investment of approximately $762 million, and would result in a total present value benefit of $4.7 billion without climate change, or $5.9 billion under a rapid warming scenario (RCP 8.5).

The integrated modeling framework we have developed leverages multiple national-scale datasets to estimate the costs and benefits of property-level adaptations to riverine flood risk. Because all of these data were generated at a CONUS scale, there are multiple sources of uncertainty that must be considered in the interpretation of our results. These include uncertainties in the modeled flood elevations that drive the fractional loss calculations; the limited availability of data documenting existing adaptations at a municipal scale; uncertainties in the cost of adaptation; and uncertainties in the future flood probabilities arising from different climate change scenarios. These uncertainties are described in detail in First Street (2020b) and Bates et al. (2021), and are summarized in more detail in the Discussion.

## 4 Discussion and Conclusions

Our analysis shows that without adaptation, the expected annual damages to residential property due to riverine flooding will increase as climate warms through the 21^st^ century. Our analysis also provides a framework for assessing the benefits of property-level adaptations to this increasing flood risk, using property-level flood risk information to simulate adaptation decisions under baseline and future climates. We show that including climate change impacts into a benefit-cost framework of flood adaptations increases the total benefits of adaptation actions made today, and that the number of properties meeting specified benefit-cost thresholds increases with climate change, for a given discount rate.

The datasets we have used for this analysis are national in scope, and provide a platform for a property-level analysis of the benefits and costs of adaptation at a national scale. In some cases, however, information like first floor elevations and building square footage had to be imputed from nearby data using statistical and spatial models (e.g., FSF 2020b; Armal et al., 2020). The inclusion of these estimates is consistent with the USACE’s HAZUS methodology as it relies on the “typical” and “likely” building characteristics in small homogenous residential locations, operationalized by census blocks. Similarly, the flood risk model that informs flood depths at each property, while physically-based, is also driven by national-scale datasets that are necessarily coarse (Bates et al., 2021; First Street, 2020b). Bates et al. (2021) summarize the uncertainties in the national-scale flood model, citing Critical Success Index scores similar to those from local-scale 2D hydrodynamic models. This suggests that at a national scale, the modeled flood depths should be similarly unbiased to those calculated using more localized hydraulic models. Finally, we compiled adaptation costs from national data, and we did not adjust construction costs to reflect local-scale market forces, which could increase or decrease costs from those used here. Each of these elements of our analysis introduces uncertainty into the property-level results. While these details may influence our results at a property level, however, our analysis provides a benchmark for quantifying the benefits and costs of national-scale adaptations to inland flood risk. Furthermore, the framework we have developed here can be easily modified to include more site-specific data and inform local adaptation decisions.

Our results should be interpreted with an understanding that the simulated adaptation responses were developed using simple decision metrics that could be applied nationally in a consistent manner. In reality, adaptation decisions will be influenced by a complex mixture of personal, government, and financial factors that will vary across space. As such, the estimates presented here should not be construed as recommending any specific policy or adaptive action. Further, additional adaptation options not included in this analysis, such as levees and riparian restoration, may be appropriate, and potentially more cost-effective, for some locales.

Because a strict benefit cost calculation discounts future changes in flood risk, our modeling shows that adaptation decisions made today, based on reliable information on current flood risk, will generally be similar to the decisions that would be made if climate change were included in the benefit-cost calculation. This finding is particularly relevant given the uncertainties inherent in climate change projections (e.g., Tebaldi et al. 2021, Meehl et al. 2020), and the near-exponential growth in the availability of “climate change services” to insurance, finance and government (e.g., Jacobs and Street, 2020; Fiedler et al., 2021). As individuals and local governments struggle with how to incorporate climate change impacts into their decision-making, it is likely that understanding current flood risk, and determining an appropriate discount rate and return on investment (i.e., benefit cost threshold) may be more important considerations than the nuances of how climate change is incorporated into the future risk equation.

There are also reasons to believe that a strict benefit-cost adaptation test may not be appropriate in all cases. First, our analysis assumes that adaptation action is taken immediately, whereas implementation of any adaptation scenario would take a large amount of political will, capital, and acceptance by property owners. Second, there is evidence that communities and property owners invest in risk reduction less than strict cost-benefit tests would predict, and that many non-economic factors like perception of risk, trust in the source of risk, and various psychological heuristics and biases affect risk reduction decisions (e.g., see Bubeck et al, 2012; Poussin et al, 2014). Use of a larger benefit-cost ratio approximates the aggregate effect of such sub-optimal property-level decisions. And finally, our strictly financial analysis does not account for the human health, emotional, and social impacts of natural disasters like flooding, which can influence both individuals’ adaptation behaviors prior to an event and the impacts from flooding after an event (e.g., Paterson et al., 2018; Buchecker et al., 2013; Siegrist and Gutscher, 2008).

We would expect private decisions about adaptation at individual properties to reflect costs and benefits to the extent the decision-maker has access to reliable and trustworthy information about those costs and benefits. However, from a social standpoint, economic cost-benefit tests may not be the preferred approach for government investment in adaptation (e.g., Kind et al., 2020). For example, inland flooding may disproportionately affect socially vulnerable populations (e.g., Osberghaus, 2021; Cutter et al., 2003), and in some cases climate change could exacerbate those inequities (e.g., Mills et al., 2019). There are strong arguments for incorporating these considerations into decision-making to ensure that future vulnerabilities to natural hazards do not fall disproportionately on subsets of the United States population.

## Supplementary Material

Supplementary Material

## Figures and Tables

**Table 1.** Summary of national flood adaptation costs and benefits under different assumed benefit cost thresholds (rows) and discount rates (columns). Values rounded to the nearest million dollars.

		Discount Rate		
		1%	3%	6%

**BCR>1**	Cost	$64,186,000,000	**$46,317,000,000**	$14,403,000,000
	Benefit No CC	$109,298,000,000	**$68,725,000,000**	$22,601,000,000
	Benefit With CC	$120,782,000,000	**$81,002,000,000**	$38,105,000,000

**BCR>2**	Cost	$10,112,000,000	**$5,466,000,000**	$2,256,000,000
	Benefit No CC	$32,335,000,000	**$16,934,000,000**	$6,878,000,000
	Benefit With CC	$38,802,000,000	**$20,782,000,000**	$9,966,000,000

**BCR>4**	Cost	$1,628,000,000	**$762,000,000**	$291,000,000
	Benefit No CC	$9,916,000,000	**$4,674,000,000**	$1,807,000,000
	Benefit With CC	$12,112,000,000	**$5,885,000,000**	$2,652,000,000
