# Supplementary material for: Climate change, riverine flood risk and adaptation for the conterminous United States
Source: Environ Res Lett. Author manuscript; Available in PMC 2021 Sep 23. (PMC8459676; doi:10.1088/1748-9326/ac1bd7)
Supplement: Supplementary Material [file NIHMS1737018-supplement-Supplementary_Material.docx]

**Supplemental Information File: Adaptation Cost Estimates**

This document summarizes the data used to estimate unit adaptation costs for dry floodproofing and elevation. Property acquisition cost was assumed to be the market value of each property, which is included in the First Street Foundation database.

Dry Floodproofing

In a dry floodproofing approach, the livable area that would be exposed to flood water is made watertight to eliminate the potential of the space being inundated in a flood situation. For this study, we assume dry floodproofing is achieved with permanent watertight coating on exterior walls of the structure. This prevents water from entering basements or crawlspaces. The watertight coating can be applied to above-ground exterior walls up to three feet above ground level. Above three feet of inundation depth involves hydrostatic pressure that many structures may not be able to withstand (FEMA, 2009) and it may start to reach the height of window sills, which are difficult to seal.

FEMA (2009) indicates that dry floodproofing is generally not appropriate for homes with basements due to the high potential for foundation walls to fail under increased hydrostatic pressure when flooded. In addition, dry floodproofing may not be effective for long duration and/or high velocity flood conditions. While such conditions are relevant to this study (e.g., floods on the largest rivers, such as the Mississippi, can last weeks, and flash floods in many urban areas involve extremely high flow velocities) these factors were not modeled in the current study. We assume dry flood proofing is feasible for any structure that does not have a basement and will eliminate flood damage for depths up to three feet above ground. It’s also important to note that FEMA may not allow dry floodproofing under floodplain management regulations, and is unlikely to reduce insurance premiums as a result of dry floodproofing for residential structures.

Unit costs for dry floodproofing depend the specific combination of techniques chosen. Table S1 shows unit costs for several alternatives (FEMA, 2009, adjusted to 2020 dollars). We include the following measures from FEMA (2009): 1) waterproof membrane, above grade only, 2) drainage lines around the perimeter of the structure, 3) one plumbing check valve per structure, 4) one sump pump per structure, and 5) six linear feet of metal flood shield per 1000 square feet of footprint. Dry floodproofing for a 1000 square foot, single story house, with a 130 foot perimeter, would total $13,513.

**Table S1**: unit costs for dry floodproofing measures used in analysis (FEMA, 2009)

| **Dry flood proofing measure** | **Cost** | **Per unit** |
| --- | --- | --- |
| Sprayed-on cement (above grade) | $23 | linear foot of wall covered |
| Waterproof membrane (above grade) | $8 | linear foot of wall covered |
| Asphalt (two coast on foundation up to 2 feet below grade) | $17 | linear foot of wall covered |
| Drainage line around perimeter of structure | $43 | linear foot |
| Plumbing check valve | $1,463 | each |
| Sump and sump pump | $2,360 | Lump sum |
| Metal flood shield | $518 | linear foot of shield surface |
| Wood flood shield | $161 | linear foot of shield surface |

Elevation

Elevating a home or structure involves lifting the structure above a design flood elevation. This is accomplished either by lifting the entire foundation and structure (for slab-on-grade foundations) or by extending foundation walls vertically (typically for structures with a basement or crawl space). Unit costs were taken from FEMA (2009). FEMA (2009) provides unit cost values for three increments of elevation. The unit cost for 2 feet of elevation was applied for all increments up to four feet, while the unit costs for 4 to 8 feet were applied for all increments of elevation above four feet (see Figure S1). The unit costs include assumption of a substantial fixed cost regardless of how much a structure will be elevated; the fixed cost covers design, regulatory requirements, and mobilization of construction equipment.

**Figure S1**: Elevation costs used in analysis for buildings with and without basements (FEMA, 2009).

Inflation adjustment

All unit costs used from the studies listed above were inflated to 2020 dollars using the Construction Price Index for single family homes from the U.S. Census (2021).

References

FEMA (2009). Homeowner’s Guide to Retrofitting. Second edition. US Department of Homeland Security: Federal Insurance and Mitigation Administration (FEMA), Washington, DC. Avaialble at: <http://www.fema.gov/library/viewRecord.do?id=1420>

U.S. Census (2021). Construction Price Indexes. Available at: https://www.census.gov/construction/cpi/ (accessed January 25, 2021).
